# An isotope signature for diffuse idiopathic skeletal hyperostosis?

**DOI:** 10.1002/ajpa.24497

**Published:** 2022-03-03

**Authors:** Laura Castells Navarro, Jo Buckberry, Julia Beaumont

**Affiliations:** ^1^ Department of Archaeology University of Exeter Exeter UK; ^2^ School of Archaeological and Forensic Sciences University of Bradford Bradford UK

**Keywords:** bone collagen, carbon, diet, diffuse idiopathic skeletal hyperostosis, DISH, nitrogen, Roman period

## Abstract

**Objectives:**

Diffuse idiopathic skeletal hyperostosis (DISH) has recurrently been associated with a rich diet (high in protein and higher trophic level foods); however, very few studies have investigated this link using carbon and nitrogen (*δ* ^13^C and *δ* ^15^N) stable isotope analysis. This paper explores the relationship between DISH and diet in two Roman urban communities by analyzing individuals with and without DISH.

**Materials and methods:**

*δ* ^13^C and *δ* ^15^N analysis carried out on collagen from 33 rib samples (No DISH: 27; early DISH: 4; DISH: 2) selected from individuals buried at the Romano‐British site of Baldock (UK), 41 rib samples (No DISH: 38; early DISH: 3) from individuals from the Catalan Roman site of Santa Caterina (Barcelona, Spain). Additionally, six faunal samples from Baldock and seven from Santa Caterina were analyzed.

**Results:**

Standardized human isotope data from Santa Caterina show high *δ* ^15^N probably associated to a diet combining terrestrial resources and freshwater fish. In contrast, isotope results from Baldock suggest a terrestrial‐based diet. Individuals with DISH do not show isotopic ratios indicative of rich diet and there is no correlation between stage of DISH development and *δ* ^13^C and *δ* ^15^N.

**Conclusion:**

The results of this study suggest that individuals with DISH followed a similar or isotopically similar diet as those individuals without DISH in Baldock and in Santa Caterina and therefore, while DISH may have been influenced by individual's dietary habits, this is not reflected in their isotopic signature.

## 1 INTRODUCTION

In bioarchaeology, diffuse idiopathic skeletal hyperostosis (DISH) has been related to a rich diet due to its clinical association to obesity and diabetes. Overall diet composition and rich diets have been investigated using stable isotope analysis, but the relationship between diet and DISH has rarely been investigated (see Section 1.3) using stable isotope analysis. This paper aims to investigate the relationship between DISH and diet by comparing the isotopic signature of individuals with and without DISH and the prevalence of DISH in two communities which we believe followed different diets—a terrestrial “British” diet, and a cereal, olive oil and vegetables‐based “Mediterranean” diet—in two urban communities from the Roman period, one from England and the other from Catalonia (Spain).

### 1.1 Diffuse idiopathic skeletal hyperostosis (DISH) in clinical and paleopathological research

Diffuse idiopathic skeletal hyperostosis (DISH) is a progressive hyperostotic condition characterized by the ossification of ligaments and tendons or adjacent structures (Mader et al., 2013, p. 9). Clinically, DISH is more prevalent in male individuals and, while all studies show higher prevalence in males than in females, the sex‐based difference in prevalence reduces in older age groups (Cassim et al., 1990; Julkunen et al., 1971; Weinfeld et al., 1997).

Traditionally, the most common manifestations of DISH are a spinal “flowing” ossification and extra‐spinal enthesopathies (Crubézy, 1989; Resnick et al., 1975; Utsinger, 1985), and its diagnosis is considered to be straight‐forward in archaeological human remains (Crubézy & Trinkaus, 1992; Rogers & Waldron, 1995). Despite this, there are several diagnostic criteria for DISH described that can result in significantly different calculated prevalence rates when applied to the same skeletal sample (van der Merwe et al., 2012) and there is little consensus with regards to the presence of extra‐spinal manifestations (ESM) in patients with DISH (Kuperus et al., 2017; Mader et al., 2013).

Clinically, DISH has been associated to obesity or high BMI (Denko & Malemud, 2006; Diederichs et al., 2011; Forestier & Rotes‐Querol, 1950; Sarzi‐Puttini & Atzeni, 2004; Utsinger, 1985), to diabetes mellitus (Daragon et al., 1995; Diederichs et al., 2011; el Miedany et al., 2000; Littlejohn & Smythe, 1981; Mader & Lavi, 2009; Sencan et al., 2005; Westerveld et al., 2014) and to hyperuricemia, dyslipemia and cholesterolemia (Denko & Malemud, 2006; el Miedany et al., 2000; Kiss et al., 2002; Miyazawa & Akiyama, 2006; Sarzi‐Puttini & Atzeni, 2004; Vezyroglou et al., 1996) suggesting that DISH patients are commonly affected by metabolic imbalances (Utsinger, 1985). The relationship between DISH and hypertension (el Miedany et al., 2000; but see Kiss et al., 2002; Mader & Lavi, 2009; Utsinger, 1985), levels of growth hormone and insulin‐like growth factors (Denko & Malemud, 2006; Sencan et al., 2005), and cardiovascular conditions (Denko & Malemud, 2006; el Miedany et al., 2000; Mader et al., 2005; Westerveld et al., 2014) has also been explored although Miyazawa and Akiyama (2006) argued that the increased risk of stroke in DISH patients could also be related to increased uremia and diabetes or obesity, which are risk factors for stroke. Several of these conditions have been linked to diets with high cholesterol, including dairy and red meats. The modern Mediterranean diet and dietary pattern, however, seems to reduce the risk of cardiovascular disease and diabetes (Forman & Bulwer, 2006; Schröder, 2007; Schwingshackl et al., 2015).

Because of the relationship between DISH, obesity, and diabetes, in paleopathology, DISH has recurrently been linked to monastic and high‐status communities. Waldron's (1985) studies on the remains found at the cemetery of Merton Priory suggested that monastic people had significantly higher prevalence of DISH than the layfolk. These results were interpreted as meaning that medieval monks ate much more than their allowance. These observations concurred, according to the author, with some contemporary records that suggested that monastic diet included significant amounts of meat and fish (Waldron, 1985). In 2001, Rogers and Waldron suggested that the “monastic way of life” characterized by the dietary characteristics previously described by Waldron (1985) would have led to obesity and type II diabetes and, in turn, to the increase predisposition to develop DISH. In a similar study, Verlaan et al. (2007) reported that 40.4% of the adult individuals buried at Abbey Court (Pandhof) Maastricht (Netherlands), and assumed to be clergymen and high‐status individuals, showed ossifications of, at least, four vertebrae and multiple ESM. Finally, in an interdisciplinary approach to investigate the validity of the stereotype of the “obese medieval monk”, Patrick (2014) concluded that monks were three times more likely to develop DISH and over five times more likely to develop one form of obesity‐related osteoarthritis compared to the layfolk (Patrick, 2014, p. 152). However, the small sample size did not allow the relationship between monastic lifestyle and obesity to be asserted (Patrick, 2014, p. 117). In contrast, Mays (2006) called for caution in the interpretation of this type of data as the studies were based on relatively small number of samples and without proper non‐monastic age‐matched control groups. Furthermore, as DISH seems to be more prevalent in older male individuals, the focus on the monastic community and its inherently sex and age bias was likely to result in an overestimated prevalence of this condition (Mays, 2006).

### 1.2 Light stable isotope analysis and dietary reconstruction

Krueger and Sullivan's (1984) model on the relationship between diet and collagen proposed that all the carbon in bone collagen originated from the protein fraction of the diet whilst the mineral component of the bone, hydroxyapatite, reflected the values of the whole diet (Jim et al., 2004). 98% of the nitrogen it is locked in proteins and amino acids, with the remaining 2% forming part of nucleic acid, urea and ammonia.

*δ* ^13^C and *δ* ^15^N values are known to increase in each step of the food chain in what is known as the “trophic level effect” (O'Connell et al., 2012). Carbon shows an increment of 1‰ from producers to consumers that is too small to be useful to evaluate the consumer's position in the food chain (Schoeninger & DeNiro, 1984; Schwarcz & Schoeninger, 1991) so this position is assessed using *δ* ^15^N values. Most studies consider that the average *δ* ^15^N enrichment between trophic levels is around 3–4‰ (Hedges & Reynard, 2007; O'Connell et al., 2012); however, in humans, this value can vary between 1.8 and 6‰ (Huelsemann et al., 2013). Nonetheless, human isotopic signature will reflect the *δ* ^13^C and *δ* ^15^N values of the protein element of the diet and will therefore allow the discrimination of the trophic level of the foodstuff consumed. Thus, in this context, “rich diet” is defined as that showing high *δ* ^15^N values, meaning that the protein came from an animal high in the food chain. However, it is worth noting that *δ* ^15^N values are known to increase in cases of physiological stress associated to growth, nutritional stress or illness (e.g., D'Ortenzio et al., 2015; O'Donoghue et al., 2021). As these factors do not seem to impact *δ* ^13^C values, the resulting in *δ* ^13^C and *δ* ^15^N signature of individuals with physiological stress does not reflect their diet.

To carry out a reliable diet reconstruction of past populations, the isotope composition of all the possible available foodstuff should be analyzed (Dufour et al., 1999; Schwarcz & Schoeninger, 1991).

### 1.3 DISH and stable light isotope analysis: Previous studies

Because of the association of DISH with monastic communities and high‐status individuals, DISH has been linked to a “rich diet,” understood as those diets high in protein and higher trophic level foods. In this sense, isotope analysis has been used to corroborate the relationship between DISH and status and therefore, it has been hypothesized that individuals with DISH would show higher *δ* ^13^C and *δ* ^15^N than individuals without DISH if they were eating a protein‐rich diet. Given that the spinal lesions associated to DISH have been suggested to take at least a decade to develop (Mader, 2008; Yaniv et al., 2014), it would be expected that differences in diet were long term and would therefore be evident in the isotopic data obtained from rib.

Müldner and Richards (2007a, 2007b) reported on the *δ* ^13^C and *δ* ^15^N of four individuals with DISH from the late medieval Gilbertine priory of St Andrew, Fishergate (York, UK). The authors found that while the *δ* ^13^C and *δ* ^15^N of individuals with DISH was similar to that of the male individuals without DISH, the affected individuals showed higher *δ* ^13^C (between −18.8 and −18.2‰) and *δ* ^15^N (between 13.2 and 15.2‰) compared to the overall male mean of −18.9‰ (*δ* ^13^C) and 13.0‰ (*δ* ^15^N). The authors argued that this suggested individuals with DISH had a high trophic level diet with animal protein and a significant input of marine resources (Müldner & Richards, 2007a, 2007b). Spencer (2008) analyzed eight late medieval monastic and non‐monastic sites and found no statistically significant relationship between the DISH status (DISH vs non‐DISH) and *δ* ^13^C. However, individuals with DISH seemed to have higher values of *δ* ^15^N than individuals without DISH, also suggesting that the individuals with DISH had a higher trophic level diet. These patterns were not observed when males and females were studied separately and the sample size for this study was too small to identify any difference between the DISH and the non‐DISH individuals (Spencer, 2008). More recently, Quintelier et al. (2014) analyzed 39 adult individuals (15 male and 14 female individuals without DISH, and 10 male individuals with DISH) buried in the post‐medieval Carmelite Friary of Aalst (Belgium) to investigate the relationship between DISH and diet using *δ* ^13^C and *δ* ^15^N analysis. Their results suggest differences in dietary patterns depending on social status but no statistically significant differences were found for *δ* ^13^C and *δ* ^15^N when comparing DISH and non‐DISH individuals. Quintelier and colleagues also found no statistically significant differences in the isotopic signature of pathological and non‐pathological bones from the same individuals affected with DISH (Quintelier et al., 2014).

### 1.4 Diet in the Roman period

#### 1.4.1 Romano‐British diet

Romano‐British rural and urban settlements show significant variation in environmental and material culture, possibly reflecting unique community traditions, location, environment, and economy as well as the influence of the Roman army, available imports and access to urban markets (King, 1999; Redfern et al., 2010). It seems also that only in urban areas, the Roman influence permeated all the social strata; in rural areas, only the aristocracy and the elites, eager the increase their status, adopted the “Roman lifestyle” (Cheung et al., 2012).

Documentary sources and archaeological data suggest that Romano‐British diet was dominated by terrestrial resources with a high presence of cattle and pig, which with poultry, eggs and wild game, possibly indicated a high‐status, high trophic‐level diet (Cool, 2006, pp. 98, 102; King, 1999; Maltby, 1997). Faunal remains from rural settlements show higher presence of sheep/goat, retaining the Late Iron Age dietary pattern rather than Roman influence (King, 2001). This suggests that the degree of “Romanisation” was determined by social differentiation that controlled access to food and new and existing dietary patterns (Redfern et al., 2010). The presence of fish, shellfish and fish sauces in Romano‐British sites is unequal and dependent on geographical location and the status of the individuals (Cool, 2006, p. 106; Locker, 2007). In Roman Britain, wheat was the most important staple food (White, 2000). Barley, oats and rye are recovered in much smaller quantities and its distribution throughout the territory is uneven (Cool, 2006, pp. 69, 77). During this period, new fruits, vegetables, and herbs were introduced in the Romano‐British diet although their distribution is dependent on the site location and population characteristics (Van Der Veen et al., 2007; van der Veen et al., 2008).

The isotope data obtained from Romano‐British sites indicate that most of the population followed a terrestrial C_3_‐plant based diet with a variable input of animal protein and, in some cases, small inputs of marine resources (Chenery et al., 2010; Chenery et al., 2011; Cheung et al., 2012; Müldner & Richards, 2007a, 2007b; Redfern, 2010; Richards et al., 1998). It is possible that the fish/shellfish signature is nevertheless masked by the overwhelming terrestrial signature if most of the protein intake was from C_3_ staple foods (Craig et al., 2009; Redfern et al., 2010). Isotopic data have also shown dietary differences between and within urban and rural communities, between social strata and, in some cases, between sexes (Chenery et al., 2011; Fuller et al., 2006; Richards et al., 1998).

#### 1.4.2 Roman Catalan, Spanish, and Mediterranean diet

Most of the dietary information from the Roman period comes from ancient texts describing the culinary customs of the wealthier classes of the society in Italy or the eastern Mediterranean area (Purcell, 2003; Wilkins, 2003). These resources should be used cautiously since the provincial population probably mixed the newly imported Roman habits with their former traditional way of life (King, 2001).

Documentary sources and archaeological and isotope data suggest that the Roman Catalan diet was centered on the Roman triad (cereals, olive oil and wine) supplemented with vegetables, legumes, and fruits (Ejstrud, 2006; Garnsey, 1999; Gómez i Pallarès, 1996). In this area, native C_4_ plants (e.g., millet and *Spartina sp*.) probably were either part of the diet or were fed to domesticated animals and thus entered the human food chain (Alonso Martínez, 2000; López‐Costas & Müldner, 2016; Tafuri et al., 2009). Following Roman trends, pig was possibly the preferred meat although local patterns with high cattle and sheep/goat can be found in the archaeological faunal remains (Colominas, 2017; Genera i Monells et al., 2010; Gómez i Pallarès, 1996; King, 1999; King, 2001). Cheese was the only dairy product widely available, and chickens, hens and eggs were also an important source of meat and protein (Faas, 2006). The status of fish is complex and ambiguous since while most species were considered to be for the poor and a sign of destitution, some marine species and seafood were a luxury, and documentary and epigraphic sources suggest a common consumption of fish (mainly in the coastal sites) (Gómez i Pallarès, 1996; Prowse et al., 2004; Prowse et al., 2005). However, isotope data do not suggest that fish and fish sauces had a significant input in the Roman diet (Gómez i Pallarès, 1996; Prowse et al., 2004; Prowse et al., 2005). In fact, most isotope data obtained from Roman sites around the Mediterranean basin has been interpreted as suggestive of a diet dominated by terrestrial C_3_ plant and complemented by some meat and possibly a small input of marine resources (Fuller et al., 2010; Lightfoot et al., 2012; Nehlich et al., 2012; Rissech et al., 2016). Thus, it seems that neither meat nor fish were considered staple foods but more a supplement to the mainly vegetarian diet (Craig et al., 2009).

#### 1.4.3 Food groups, obesity, diabetes, cardiovascular diseases, and DISH

The authors are not aware of specific evidence for obesity for Roman Catalonia or Britain. Furthermore, Bradley (2011) argues that in the Roman period, obesity, corpulence and emaciation were not major themes in ancient art and the body representations answered to an honorific, standardized or idealized canon.

For this reason, the focus to explore these diseases in the Roman period was on attempting to identify those food groups that increase the risk of developing obesity, diabetes and cardiovascular diseases and that would have been available during the Roman period. These include refined grains (i.e., white bread) and red meats (Forman & Bulwer, 2006; Knowler et al., 2002; Ley et al., 2014). Plant‐based fats found in vegetable oils, nuts, and seeds, would have also been available but are associated with a lower risk in developing diabetes (Knowler et al., 2002; Ley et al., 2014). Dairy products have been shown to moderately reduce the risk of diabetes (Ley et al., 2014). The relationship between fish and shellfish and diabetes seems to be related to how the fish is cooked and consumed (Ley et al., 2014), although it is worth noting that diets high in fish and fish oils (high in omega‐3 fatty acids) reduce cardiovascular diseases (Forman & Bulwer, 2006). As this group of diseases have been associated with DISH (Denko & Malemud, 2006; el Miedany et al., 2000; Mader et al., 2005; Westerveld et al., 2014), a diet rich in fish may not result in the development of DISH. Finally, while high *δ* ^13^C and *δ* ^15^N values have been linked to rich diet and, specifically, to a meat‐rich diet, a diet rich in fish may lead to high *δ* ^13^C and *δ* ^15^N values but would most likely not have led to obesity and DISH. We hypothesize that Romano‐British diet—with a reliance on red meat—could have increased the risk of obesity and all its co‐morbidities, including DISH, and isotopically would be characterized by high *δ* ^15^N values. Whilst the Mediterranean diet, which is not associated with obesity, heart disease and diabetes today (Buckland et al., 2008; Forman & Bulwer, 2006; Schröder, 2007), might lead to a lower prevalence of DISH and would be characterized by low *δ* ^15^N values.

## 2 METHODS

### 2.1 Site and sample selection

The Romano‐British settlement of Baldock, located in North Hertforshire, was discovered in 1925 and excavated in several campaigns between then and 1994 (Burleigh et al., 1978, p. 9). The excavations revealed that Baldock, in the Chilterns, had emerged as an important oppidum in the early first century BC during the late Iron Age (Thompson, 2015). Roman Baldock was a large settlement in south‐east Britain located at the crossroads of Icknield Way, which runs east–west, and Stane Street which connected it to major regional centers such as Verulamium (St Albans), London and Colchester. It exhibits characteristics of Roman urban settlement such as planned layout and monumental buildings but as it does not seem to have had a fort, so Roman Baldock is best described as “nucleated settlement” or village. Its population seem to have been largely agriculturally engaged but there is also presence of small‐scale light industry and crafts. The Late Roman burial site of BAL‐1 (known as the California cemetery) is located at the north‐west of the settlement and dates to the period ca. ad 175–550 (Burleigh et al., 1978). Thirty‐three adult human rib fragments and six faunal samples from this site were selected light stable isotope analysis (Tables 1A and 1C).

The cemetery in Santa Caterina—Av. Francesc Cambó was first identified in two interventions in 1984 and in 1986 directed by M.T. Miró and A. Oliver and J.O. Granados, respectively; however, it was excavated between 1999 and 2002 (Aguelo Mas et al., 2001). *Colonia Iulia Augusta Fauentia Paterna Barcino* was founded in 15–5 bc on top of a hill and limited by two rivers. During the first centuries, this walled urban center developed an important suburban activity. At some point in the fourth century, it seems the city was re‐fortified and the necropolis of Santa Caterina appeared at the site of the abandoned *suburbium* outside the city walls but close to the roads accessing the city (Aguelo i Mas et al., 2005, p. 16). According to the stratigraphy, the necropolis was used between the fourth and the sixth centuries AD. Forty‐one adult human rib fragments and seven faunal samples from this site were selected for light stable isotope analysis (Tables 1B and 1C).

The individuals from both sites were selected from their respective original sample as follows: all individuals were adults, had a rib available for sampling and to ensure all ages and both sexes were represented. To allow meaningful comparison, these two communities were selected because they had both been described as both lay and urban. Furthermore, to reduce the potential bias in diet linked to status and lifestyle high status individuals—identified using burial characteristics—were not included in the sample.

### 2.2 Osteological analysis

Sex assessment was attempted once the individual had been estimated to be skeletally adult as established by epiphyseal fusion (late fusion epiphyses were not considered for this classification). The morphology of the pelvis (Klales et al., 2012; Phenice, 1969) and of the skull (Walker, 2008) were evaluated. If the pelvic and skull features were not coherent, the pelvic features were given preference as, while both areas have high accuracy rates, the function‐related pelvic dimorphism makes this area more reliable for sex assessment (Mays & Cox, 2000, p. 120). Age estimation of the adult individuals was carried out by applying transition analysis (Boldsen et al., 2002) using a combination of pubic symphysis, auricular surface and ectocranial suture closure features. To process the data, the ADBOU Age Estimation software developed by Boldsen et al. (2002) was used. This method was chosen because it avoids the age‐mimicry effect and provides a better age estimate than traditional age estimation methods (Bullock et al., 2013).

### 2.3 Diagnosis of diffuse idiopathic skeletal hyperostosis (DISH)

**FIGURE 1:**
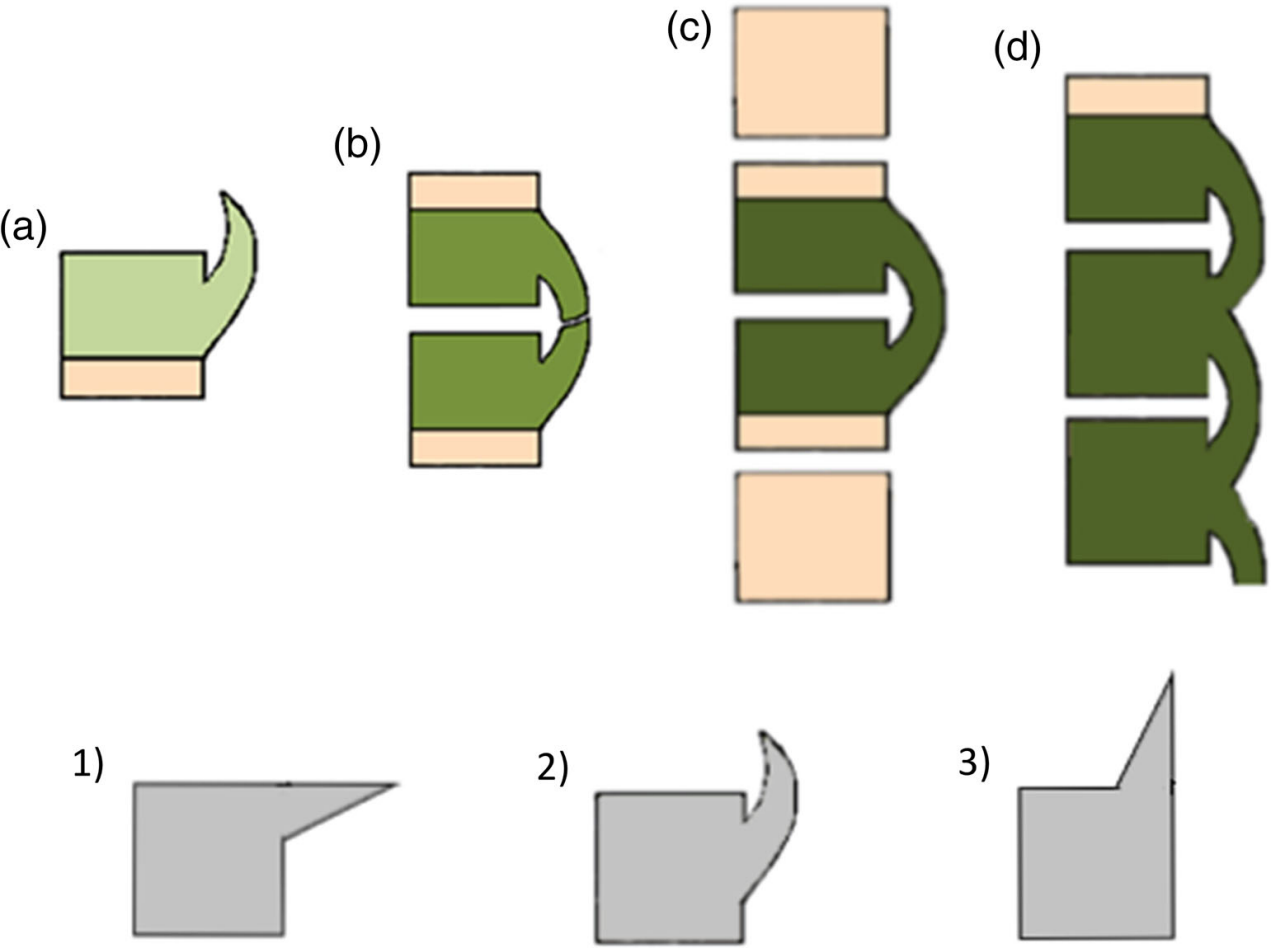
Diagrammatic representation of lesions and stages of DISH. Top row: (a) Stage 1, isolated outgrowths; (b) Stage 2, touching/interlocking outgrowths; (c) Stage 3, one complete bridge between two adjacent vertebrae; (d) Stage 4, >2 vertebrae involved in the ankylosis. Bottom row: Location and orientation of (1) vertebral outgrowths in DISH, of (2) osteophytes in discarthrosis, and of (3) syndesmophytes in ankylosing spondilitis

All the individuals analyzed conformed to an inclusion criterion aimed at standardization of the sample and prevent bias in the prevalence of DISH. This criterion indicated that: only adult with well‐preserved anterior portions of at least three vertebral bodies from the lower half of the thoracic portion of the spine would be included in the sample. DISH was diagnosed using the criteria described by Castells Navarro and Buckberry (2020) that includes the early stages of development of the disease. In short, Stage 1 is characterized by isolated outgrowths at the thoracic spine (can also be seen at the lumbar level) most often unaccompanied by disc degeneration; when isolated outgrowths are found at adjacent vertebrae, these do not touch or interlock. Stage 2 is characterized by touching or interlocking outgrowths in adjacent thoracic and/or lumbar vertebrae usually unaccompanied by disc degeneration. Stage 3 is characterized by the presence of one complete osseous bridge between two adjacent vertebrae. The apophyseal joints are not affected, the intervertebral disc space is retained and in very rare cases the vertebral endplates show disc degeneration. Finally, in stage 4 more than two vertebrae are involved in the ankylosis, the apophyseal joints are not affected, the intervertebral disc space is retained and in very rare cases the endplate of the affected vertebrae show disc degeneration. Stages 1 to 3 represent early or pre‐DISH whereas stage 4 represents DISH (Figure 1).

Early DISH lesions are clearly distinguishable from those of discarthrosis due to their internal structure, relation to the endplate and orientation. As already observed by Forestier & Rotes‐Querol (1950), the structure of DISH lesions at all stages of development (when it was possible to observe the internal structure, for example in outgrowths with post‐mortem breaks) mirror that of normal bone: an external well‐organized and smooth cortical bone and an internal trabecular bone, as if this ossification were an extension of the vertebral body itself. Unlike vertebral osteophytes, DISH‐related enthesophytes are, at all stages of development, usually rooted at the central third or at the interphase between the central and the upper or lower sections of the vertical sides of the vertebral body. And finally, DISH‐related enthesophytes are vertically oriented and curve around the intervertebral disk (Castells Navarro & Buckberry, 2020).

### 2.4 Carbon and nitrogen stable isotope analysis

For stable isotope analysis, rib samples were selected because recent research has indicated no difference in the isotope data obtained from rib and DISH lesions (Quintelier et al., 2014), and sampling of pathological lesions should be avoided where possible (Mays et al., 2013). Collagen was prepared using the modified Longin method (Brown et al., 2006; O'Connell & Hedges, 1999). Approximately 500 to 700 mg of bone was sampled, submerged in 0.5 M hydrochloric acid and stored in the fridge until complete demineralization. Samples were then rinsed with de‐ionized water and placed in sealed tubes with a pH 3 hydrochloric acid at 70°C for 48 h to allow the collagen fibrils to go into solution. The bone was then filtered with Ezee Filter separators and each sample was then frozen at −36°C for at least 24 h and freeze‐dried for 48 h to remove any remaining water. Each sample was measured in duplicate, with 0.3–0.6 mg of collagen weighed into a tin capsule. The samples were then combusted in a Thermo Flash EA 1112 and the separated N_2_ and CO_2_ was introduced to a Delta plus XL via a Conflo III interface. The results are expressed using the delta notation (*δ*) in parts per thousand (per mil; ‰) relative to the international standards: marine limestone (VPDB) for carbon and AIR for nitrogen isotope ratios. When calibrated against international and laboratory standards, the analytical error was determined at ±0.2% (1 SD) or better.

Certified (primary) reference materials (IAEA, International Atomic Energy Agency, IAEA Laboratories, Seibersdorf, Reference Materials Group) were used in order to calibrate in house (or internal) laboratory standards. The approach taken for measuring unknown samples was to analyze both certified materials and in‐house standards, along with the unknown samples in the same run. Standards were chosen that encompass the range of expected values from the samples and interspersed throughout the run bracketing the unknown samples and allowing the identification of any instrument drift. The analytical error from instrument runs of standards was determined as ≤0.19 (1 SD) for nitrogen and ≤0.10 (1 SD) for carbon (Beaumont et al., 2013).

### 2.5 Analysis of data

Non‐parametric Mann Whitney tests were performed to explore the relationship between status of DISH (presence and absence) and *δ* ^13^C and *δ* ^15^N values considering Baldock's and Santa Caterina's data separately as well as a combined sample. Human isotopic data were standardized by subtracting the average of the corresponding fauna to each of the human data points.

## 3 RESULTS

Seventy‐four adults from Baldock and 87 from Santa Caterina were analyzed for age, sex and DISH status. Seven individuals at Baldock and six individuals at Santa Caterina had DISH‐related lesions, giving a prevalence of 9.5% and 6.9%, respectively (Castells Navarro & Buckberry, 2022). There is no statistically significant difference in the prevalence of DISH at the two sites (Fisher's exact test *p*‐value = 0.576).

The age and sex data for the individuals sampled for isotope analysis are given in Tables 1, 2, and 3. Six individuals from Baldock and three individuals from Santa Caterina had DISH‐related lesions. In this sample, DISH is more prevalent in old male individuals (*n* = 6); however, two middle adult males and an old female individual were also affected. These demographics agree with the consensus that DISH is more prevalent in male individuals and that the prevalence increases with age (Julkunen et al., 1971; Weinfeld et al., 1997). Of the individuals with DISH lesions selected for this study, three individuals were identified as being at stage 1, four at stage 2, and two at stage 4 (Table 1, 2, and 4). Only the two individuals in stage 4 (Baldock F92 and 1122) would be diagnosed with DISH; the other stages represent early, pre‐DISH lesions as described by Castells Navarro and Buckberry (2020).

**TABLE 1:** Individuals with and without DISH selected for isotope analysis from the Romano‐British site of Baldock

Ind. ID	Sex	Age (95%)	DISH	δ^15^N‰	δ^13^C‰	Amt%N	Amt%C	C:N
Bal F18 L10	M	33.8 (26.3–44)	N	9.2	−19.9	16.5	45.7	3.2
Bal F466	M	39.4 (27.1–73.5)	N	9.2	−19.5	15.5	43.3	3.3
Bal F475 L2	M	73.2 (50.7–88.1)	N	11.0	−19.2	16.5	45.9	3.3
Bal F610 L1	F	26.7 (20.7–35.5)	N	10.0	−20.1	17.4	47.9	3.2
Bal F644	F	74.5 (38.3–90.3)	N	9.9	−20.9	17.7	49.2	3.2
Bal 883	M	31.1 (25.1–39.1)	N	10.0	−20.5	17.3	47.4	3.2
Bal 1022	F	63.5 (33.9–84)	N	9.2	−20.7	17.3	48.1	3.2
Bal 1028	F	32.3 (22.6–50.0)	N	9.2	−19.9	28.7	77.8	3.2
Bal 1040	F	61.8 (32.6–86.6)	N	9.2	−19.9	17.5	48.1	3.2
Bal 1077	F	75.1 (54.1–89.2)	N	9.7	−19.0	16.4	45.7	3.3
Bal 1090	M	48.6 (33.5–74.2)	N	9.6	−20.0	16.5	45.8	3.2
Bal 1107	F	71.0 (45.8–87.4)	N	10.2	−19.9	18.1	49.7	3.2
Bal 1174	M	28.9 (28.9–84.4)	N	8.5	−19.9	17.8	49.0	3.2
Bal 1203	M	72.5 (47.4–87.9)	N	10.3	−20.5	18.0	49.6	3.2
Bal 1237	M	75.9 (49.5–90.6)	N	11.2	−20.9	17.7	49.1	3.2
Bal 1263	M?	A	N	10.2	−21.1	17.2	48.1	3.3
Bal 1281	F	73.1 (49.4–88.6)	N	8.0	−20.2	13.3	36.7	3.2
Bal 1319	F	68.6 (44.8–85)	N	9.8	−20.1	16.8	46.0	3.2
Bal 1320	M	41.4 (29–67.8)	N	9.7	−20.4	15.1	42.3	3.3
Bal 1342	U	64.1 (33.1–84.9)	N	10.6	−20.6	18.3	50.2	3.2
Bal 1372	M	70.6 (44.5–87.1)	N	9.9	−20.7	18.3	49.3	3.1
Bal 1391	F	62.5 (27.3–85.5)	N	8.9	−20.3	17.9	49.0	3.2
Bal 1446	F	39.2 (27.1–61.2)	N	9.9	−20.7	17.8	48.4	3.2
Bal 1447	F	38.3 (21.4–71.8)	N	8.8	−20.4	17.2	46.8	3.2
Bal 1480	M	29.9 (21.5–45.6)	N	10.2	−19.8	16.2	44.8	3.2
Bal 2601	F?	82.1 (65.2–93.6)	N	10.1	−21.0	19.2	52.1	3.2
Bal 3644	M	37.5 (28.2–54)	N	10.5	−20.1	16.5	45.7	3.2
**Mean**				**9.7**	**−20.2**			
Bal 1072	M	47.0 (32.5–75.1)	1	9.9	−20.0	17.7	48.5	3.2
Bal 1049	M	75.6 (34.6–91.4)	2	9.7	−19.3	17.5	48.3	3.2
Bal 1374	M	74.3 (29.1–90.8)	2	9.4	−20.0	17.5	48.3	3.2
Bal 2225	M	45.8 (32–72.5)	2	9.7	−20.2	18.2	50.2	3.2
Bal F92	M	79.7 (60.6–92.2)	4	10.1	−20.1	16.3	45.0	3.2
Bal 1122	F	80.0 (61.8–92.4)	4	10.1	−20.1	17.1	47.5	3.2
**Mean**				**9.8**	**−20.0**			
**Overall mean**				**9.8**	**−20.2**			
**SD**				**0.67**	**0.50**			

*Note*: Sex: M: male; M?: possible male; F: female; F?: possible female. Age: A: adult. DISH: N: no DISH; 1–4: stages of DISH development as defined by Castells Navarro and Buckberry (2020).

**TABLE 2:** Individuals with and without DISH selected for isotope analysis from the Roman site of Santa Caterina

Ind. ID	Sex	Age (95%)	DISH	δ^15^N‰	δ^13^C‰	Amt%N	Amt%C	C:N
010/04‐T3	M	38.1 (21.0–81.9)	N	10.5	−20.0	12.7	35.2	3.2
010/04‐T14	M	76.6 (40.3–91.5)	N	10.3	−19.6	12.9	35.7	3.2
010/04‐T15	M	72.4 (40.7–89.1)	N	9.8	−19.9	11.2	31.2	3.2
010/04‐T18	M?	70.8 (21.9–89.3)	N	9.2	−20.0	10.4	29.7	3.3
002/00‐706	M	47.6 (24.0–83.2)	N	9.7	−16.2	12.8	35.2	3.2
002/00‐707	F	54.1 (34.0–78.4)	N	9.7	−19.3	12.7	35.0	3.2
002/00‐708	F?	26.7 (26.7–45.3)	N	9.7	−19.2	13.9	38.2	3.2
002/00‐709	M	76.0 (52.5–90.3)	N	10.2	−18.8	15.7	43.4	3.2
002/00‐710	M	15.0 (15.0–21.4)	N	8.8	−19.1	13.4	37.1	3.2
002/00‐719	M	71.7 (71.7–90.4)	N	9.9	−19.5	13.5	37.2	3.2
002/00‐721	F?	18.3 (18.3–31.4)	N	9.4	−19.4	13.2	36.6	3.2
002/00‐725	F	66.3 (40.6–85.6)	N	9.8	−19.3	13.7	37.6	3.2
002/00‐729	F	74.6 (47.9–89.9)	N	9.9	−19.6	13.4	37.3	3.3
002/00‐731	M	46.5 (32.1–74.4)	N	9.8	−19.4	12.6	34.7	3.2
002/00‐732	F	32.7 (24.5–46.3)	N	9.7	−19.3	12.7	35.1	3.2
002/00‐733	F	35.4 (25.8–51.5)	N	9.9	−19.6	13.4	37.3	3.2
001/01‐748	F?	77.3 (44.6–92.0)	N	9.3	−20.2	12.1	33.7	3.2
001/01‐755	F	71.4 (47.5–87.7)	N	9.6	−18.8	12.3	34.2	3.2
001/01‐758	F	15.0 (15.0–30.1)	N	10.2	−19.1	10.2	27.9	3.2
072/86‐A1.124	M	75.2 (49.1–90.1)	N	10.1	−18.9	13.1	36.2	3.2
072/86‐B1.93	F	76.8 (46.5–91.3)	N	10.3	−19.2	10.9	30.2	3.2
072/86‐B1.119	M	35.0 (27.6–46.2)	N	10.1	−19.1	11.5	32.2	3.3
072/86‐B1.123	F	66.0 (42.8–83.8)	N	10.1	−19.1	12.9	35.7	3.2
072/86‐B1.148	M	50.5 (24.6–84.8)	N	9.5	−19.2	12.9	35.8	3.2
072/86‐B2.50	M	63.3 (31–86.7)	N	11.1	−18.7	13.2	36.6	3.2
072/86‐B2.57	F	58.3 (29.9–83.2)	N	9.9	−19.3	13.3	36.8	3.2
072/86‐B2.62	M	39.2 (30.3–54.9)	N	10.9	−18.6	13.6	37.5	3.2
072/86‐B3.21	F	60.8 (37.5–81.1)	N	10.6	−19.1	13.1	36.8	3.3
072/86‐B3.59	M	37.3 (27.6–54.1)	N	11.1	−18.8	13.2	37.3	3.3
072/86‐B3.67	F	34.3 (25.2–50.3)	N	10.6	−19.0	12.7	35.9	3.3
072/86‐B3.87	M	30.5 (25.6–37.0)	N	10.7	−19.0	13.5	37.4	3.2
018/01‐T15	M	35.2 (23.3–63.1)	N	8.6	−19.6	12.5	35.0	3.3
018/01‐T25	M	36.6 (27.8–51.4)	N	8.8	−19.6	12.2	34.4	3.3
018/01‐T29	F	42.1 (28.7–64.3)	N	9.8	−19.6	12.0	33.3	3.2
201/05‐UF1	F	51.2 (32.8–77.3)	N	10.1	−19.7	15.2	42.9	3.3
201/05‐UF19	F	50.8 (25.3–81.3)	N	10.0	−19.6	17.0	46.6	3.2
201/05‐UF28	F	34.9 (25.6–50.8)	N	9.4	−19.6	16.2	45.1	3.2
162/06‐UF06	M	35.1 (27.9–45.7)	N	9.8	−18.9	15.5	43.6	3.3
**Mean**				**9.9**	**−19.2**			
002/00‐727	M	75.6 (54.5–89.6)	1	10.2	−19.3	14.3	40.2	3.3
001/01‐754	M	77.0 (21.6–110)	1	9.1	−19.0	12.6	34.8	3.2
001/01‐756	M	72.8 (44.9–88.9)	2	8.6	−19.3	11.5	31.9	3.2
**Mean**				**9.3**	**−19.2**			
**Overall mean**				**9.8**	**−19.2**			
**SD**				**0.61**	**0.61**			

*Note*: Sex: M: male; M?: possible male; F: female; F?: possible female. DISH: N: no DISH; 1–4: stages of DISH development as defined by Castells Navarro and Buckberry (2020).

**TABLE 3:** Animal samples used as isotopic reference

Site	Code	Animal	δ^13^C‰	δ^15^N‰	Amt%C	Amt%N	C:N
Baldock	BAL1480‐1	Sheep/goat	−22.2	8.0	50.1	18.2	3.2
	BAL1480‐2	Sheep/goat	−21.4	4.7	33.6	12.0	3.3
	BAL1480‐3	Sheep/goat	−21.4	5.5	44.1	16.0	3.2
	BAL2322‐1	Cattle	−21.8	7.9	45.0	16.1	3.3
	BAL1480‐2	Cattle	−21.7	8.3	47.7	17.3	3.2
	BAL1480‐3	Cattle	−22.2	7.8	47.4	16.7	3.3
	**Mean**		**−21.8**	**7.0**			
Santa Caterina	018‐01‐450	Cattle	−20.1	3.8	40.8	14.8	3.2
	018‐01‐456	Pig	−20.1	3.7	38.3	13.8	3.2
	137‐05‐81.1	Sheep/goat	−19.6	7.9	39.7	14.4	3.2
	137‐05‐81.2	Sheep/goat SA	−19.3	6.7	36.8	13.1	3.3
	137‐05‐100	Goat	−20.1	3.8	45.8	16.7	3.2
	137‐05‐123	Cattle	−22.0	2.6	37.5	13.6	3.2
	137‐05‐125	Dog	—	—	—	—	—
	**Mean**		**−20.2**	**4.8**			

**TABLE 4:** Individuals with DISH selected for stable isotope analysis

Period	Site	Ind.	Sex	Age		Vertebrae affected			DISH stage
				MLE (95%)	Y/M/O	Isolated	Interlocking	Ankylosed	
Romano‐British	Baldock	1072	M	47.0 (32.5–75.1)	M	L2, L4	—	—	1
	Baldock	1049	M	45.8 (32–72.5)	M	—	T8‐T12	—	2
	Baldock	1374	M	74.3 (29.1–90.8)	O	T8, T10	T5‐6, T8‐10, L2‐3	—	2
	Baldock	2225	M	75.6 (34.6–91.4)	O	T10	T12‐L1	—	2
	Baldock	F92	M	79.7 (60.6–92.2)	O	L3	T7‐9	T9‐12	4
	Baldock	1122	F	80.0 (61.8–92.4)	O	—	C6‐7, T1‐2, T3‐6, T7‐10, T11‐12	T6‐7, T10‐11	4
Roman	SC	001‐01‐754	F	77.0 (21.6–110)	O	T1	—	—	1
	SC	002‐00‐727	M	75.6 (54.5–89.6)	O	T7	—	—	1
	SC	001‐01‐756	M	72.8 (44.9–88.9)	O	—	T7‐8	—	2

*Note*: Site: SC: Santa Caterina; Sex: M: male; F: female. Age: MLE (95%) maximum likely estimate age as indicated in the output of the transition analysis software at 95% likelihood; Y: young adult (age‐at‐death: c.20–39.9); M: middle adult (age‐at‐death: c.40–59.9); O: old adult (c. age‐at‐death: >60) DISH stage: 1–4 stages of DISH development as defined by Castells Navarro and Buckberry (2020).

### 3.1 Light stable isotope results from Baldock and Santa Caterina

No collagen was obtained from the dog sample from Santa Caterina, and this sample is not considered further. The C:N ratios in all human and the remaining animal samples are 3.2 or 3.3 (Tables 1, 2, and 5) suggesting that collagen preservation was optimal and the values could be confidently used (van Klinken, 1999).

**TABLE 5:** Summary of the individuals selected for isotope analysis

		Young adult	Middle adult	Old adult	Adult	Combined
Baldock	Males	6	4	7	1	18
	Females	4	0	10	0	14
	N/A	0	0	1	0	1
Santa Caterina	Males	9	3	10	0	22
	Females	7	5	7	0	19
	N/A	0	0	0	0	0

*Note*: Age: young adult (c.18–39.9 years); middle adult (c.40–59.9 years); old adult (c.60+ years old); A: adult; N/A: not assessable.

**FIGURE 2:**
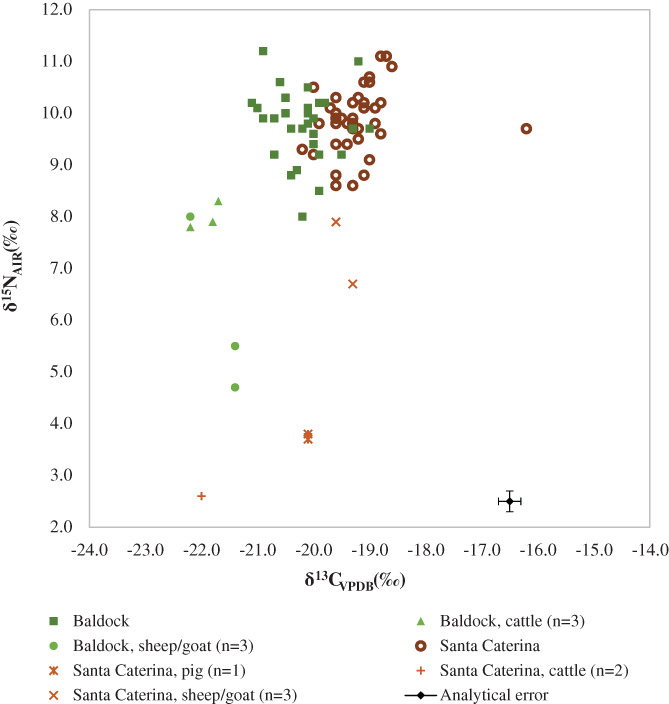
Isotope data of the human and animal remains from the Romano‐British site of Baldock and the Roman Catalan site of Santa Caterina

The *δ* ^15^N obtained from the 33 individuals from Baldock range between 8.0‰ and 11.0‰, with a mean of 9.8%, and the *δ* ^13^C range from −21.1‰ and −19.0‰ with a mean of −20.2‰ (Table 1). The *δ* ^15^N obtained from faunal samples range between 4.7‰ and 8.8‰ with a mean of 7.0‰ (1.8‰ below the average human value) while the *δ* ^13^C range between −22.2‰ and −21.4‰ with a mean of −21.8‰ (1.6‰ below the average human value) (Table 5). These results suggest that the Baldock sample generally followed a terrestrial diet. Overall, most individuals either followed a similar diet or a different but isotopically equivalent diet, with some individual variation within the group (Figure 2).

In comparison, the *δ* ^15^N obtained from the 41 individuals from Santa Caterina range between 8.6‰ and 11.0‰, with a mean of 9.8%, and the *δ* ^13^C range from −20.2‰ to −16.2‰ with a mean of −19.2‰ (Table 2). The *δ* ^15^N obtained from faunal samples range between 2.6‰ and 7.9‰ (5.0‰ below the average human value) with a mean of 4.8‰ while the *δ* ^13^C range between −22.0‰ and −19.3‰ with a mean of −20.2‰ (1.0‰ below the average human value) (Table 5). These values suggest that this sample mostly followed a terrestrial diet and the small ranges in *δ* ^13^C and *δ* ^15^N indicate that most individuals in this site consumed a similar diet or followed different but isotopically equivalent diets. Comparing the human results to the animal baseline obtained from the same site and accepting the 3‰–5‰ increment between steps of the food chain, the sample from Barcelona seem to have been either heavily reliant on sheep meat (mean *δ* ^15^N difference: 2.6‰) or have followed a more varied diet containing cattle, pig or goat meat (mean *δ* ^15^N difference: 6.4‰) possibly supplemented with freshwater fish. Individual 002‐00‐706 has bone collagen *δ* ^15^N within the population range (9.7‰) but the carbon value, at −16.2‰, and is therefore an outlier when compared to the population mean. This individual was assessed as male, with an estimated age of 47.5 (age range: 24.0–83.2 years) and with no pathologies other than linear enamel hypoplasia.

When compared, initially, the isotope data obtained from the Catalan and British individuals cluster together, which would normally be interpreted as suggesting that these individuals could have followed either similar terrestrial‐based diet or different but isotopically similar diets (Figure 2). The mean *δ* ^15^N are similar when comparing the samples from Santa Caterina and that of Baldock (9.9‰ and 9.8‰), and the mean *δ* ^13^C of the sample from Santa Caterina seems to be higher only by 1‰ compared to the Baldock's value. However, the faunal isotope data from Santa Caterina and Baldock differs, with fauna from Baldock measured with higher *δ* ^15^N and lower *δ* ^13^C compared to the fauna from Santa Caterina (Figure 2). It is also worth noting the 1.6‰ difference in the mean *δ* ^13^C in the faunal remains from Baldock (*δ* ^13^C: −21.8‰) and Santa Caterina (*δ*13C: −20.2‰). This difference could suggest that the animals from Santa Caterina consumed C_4_ plants and therefore that the slightly higher *δ* ^13^C observed in the Catalan human remains could be related to the indirect incorporation of C_4_ plants in the diet.

**FIGURE 3:**
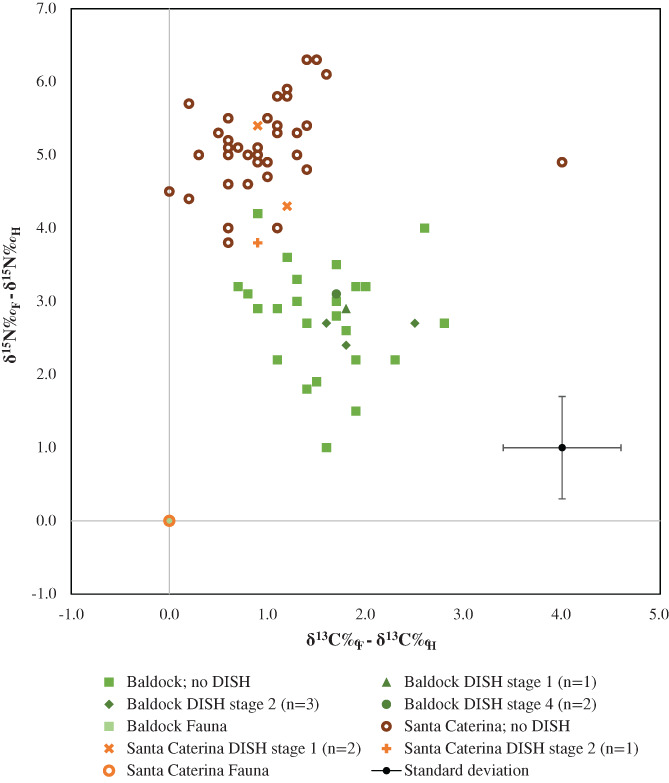
Standardized isotope data from the human remains from Baldock and Santa Caterina

We standardized the data by reducing the human *δ* ^13^C and *δ* ^15^N values by the average faunal values for the same site (Figure 3). This reveals that, once controlling for the animal baseline, that the *δ* ^13^C are very similar (difference of 0.63‰ between Santa Caterina and Baldock's samples), supporting the hypothesis that the C_4_ dietary component in Santa Caterina is a result of animal foddering, with the exception of Santa Caterina 002‐00‐706, an outlier who may have consumed C_4_ plants directly, or may have eaten meat and dairy products from animals with more C_4_ plants in their diet. A similar isotopic pattern had been observed in Roman‐era individuals from Galicia (López‐Costas & Müldner, 2016).

The striking difference in the standardized data from the two sites is the difference in *δ* ^15^N, with the average value for Santa Caterina 2.4‰ higher than the Baldock average. This suggests that, once controlling for the differences in the animal baseline, the Santa Caterina individuals were eating produce from higher up the food chain, probably indicating a reasonably sized fish‐based component in the diet. As there was little difference *δ* ^13^C isotope values, this suggests a freshwater fish component rather than marine (Hedges & Reynard, 2007). The alternate hypothesis is that the individuals from Santa Caterina were under long‐term physiological stress, resulting in a state of catabolism and increased *δ* ^15^N values with no impact on *δ* ^13^C. The latter hypothesis seems less likely, given that these individuals span a period of 200 years, all died at different ages, and that rib turnover reflects the last 3–5 years of life. Previously, physiological stress has been identified in non‐adults with rapid bone turnover who died during the Irish Potato Famine, but not in adults from the same cemetery (Beaumont et al., 2013).

### 3.2 Stable isotope results from individuals with DISH

The individuals from the Romano‐British and Roman Catalan samples were combined to investigate whether there is any relationship between DISH status and *δ* ^13^C and *δ* ^15^N. For the individuals *without* DISH (*n* = 65), the *δ* ^15^N range between 8.0‰ and 11.1‰, with a mean of 9.8%, and the *δ* ^13^C range from −21.1‰ and −16.2‰ with a mean of −19.6‰. If the outlier from Santa Caterina is removed, the mean *δ* ^13^C and *δ* ^15^N are 9.9‰ and −19.7‰. For the individuals *with* DISH (*n* = 9), the *δ* ^15^N range between 8.6‰ and 10.2‰, with mean of 9.6%, and the *δ* ^13^C range from −20.1‰ and −19‰ with mean of −19.7‰. These results show that there is no observable difference in *δ* ^13^C and *δ* ^15^N when comparing individuals with and without DISH within the same population. As the data were not normally distributed, non‐parametric tests were undertaken. This statistical analysis shows no significant difference in the *δ* ^13^C and *δ* ^15^N values of individuals with and without DISH when the sample is considered as a whole (Mann Whitney U Test; *δ* ^13^C *p* = 0.769; *δ* ^15^N: *p* = 0.359), nor when the samples from Baldock and Santa Caterina are considered separately (Mann Whitney U Test; Baldock: *δ* ^13^C *p* = 0.281, *δ* ^15^N: *p* = 0.946; Santa Caterina: *δ* ^13^C *p* = 0.662, *δ* ^15^N: *p* = 0.211). For this statistical analysis, the outlier from Santa Caterina was excluded, as the standardized data (Figure 3) has already shown that their diet was different to the other individuals from the same location. Although the sample of individuals with DISH is small, this suggests that the individuals with and without DISH followed either the same diet or isotopically similar diets. More specifically, *δ* ^13^C and *δ* ^15^N for all individuals with DISH cluster very close together suggesting no significant link between severity of the disease and type of diet as indicated by stable isotope analysis (Figure 3).

## 4 DISCUSSION

### 4.1 Interpretation of the stable isotope results from Baldock and Santa Caterina

The *δ* ^13^C and *δ* ^15^N of the individuals and the animal baseline from Baldock is comparable to other Romano‐British sites like Albert Road, Cotswold Community, Dorchester, Horcott Quarry, Old Vicarage, the Late Romano‐British individuals from the main cemetery and those buried in the mausolea from Poundbury Camp, Queensford Farm and Dorchester (Cheung et al., 2012; Fuller et al., 2006; Redfern et al., 2010; Richards et al., 1998) (see Table S1). Archaeological and documentary sources suggest that the Romano‐British population consumed freshwater and marine resources. However, it is possible that if fish was not a significant source of protein, the strong terrestrial signal observed in the individuals from Baldock could be masking the fish signature (Redfern et al., 2010). Nevertheless, most of the protein was from terrestrial origin and while it is not clear how much meat these individuals may have consumed. Research suggests that the variability in environmental and material culture may reflect unique community traditions, location, environment and economy as well as the influence of the Roman army, available imports and access to urban markets (King, 1999; Redfern et al., 2010) and, as Roman soldiers possibly consumed meat daily (Davies, 1971, p. 126), it is possible that, even in small quantities, meat was regularly consumed by the Romano‐British population; a dietary pattern that may have influenced the prevalence of DISH.

Stable isotope data from the human and animal remains from Santa Caterina suggest a Roman diet that was similar to that followed by the Roman population from Sagalassos and the Roman community from Croatia (Fuller et al., 2012; Lightfoot et al., 2012). However, the mean *δ* ^13^C and *δ* ^15^N for Santa Caterina appears marginally lower when compared to a second cluster of data from Carrer Ample (Barcelona), Mallorca, Ibiza, the Italian site of Isola Sacra and II from Velia (Craig et al., 2009; Fuller et al., 2010; Garcia et al., 2004; Prowse et al., 2004; Prowse et al., 2005; Rissech et al., 2016) (see Table S2). When comparing the human data to the animal baseline obtained from the same site, the diet of the individuals from Barcelona seem to have been based on foods like cattle and pig and regularly supplemented with anadromous fish (Chenery et al., 2010; Müldner & Richards, 2007a, 2007b). This would agree with the zooarchaeological data and the contemporary documentary sources. The significant inter‐personal variation regarding the *δ* ^15^N observed in the new data reported in this study possibly indicates that the consumption of marine resources represents individual dietary choices.

Santa Caterina individual 002‐00‐706, whose *δ* ^13^C and *δ* ^15^N were 9.7‰ and −16.2‰, respectively, has significantly higher *δ* ^13^C compared to the population mean. While the *δ* ^15^N still suggest a mainly terrestrial diet, the *δ* ^13^C resembles the data observed in the individuals from A Lanzada (northwest of Spain) that was interpreted as suggestive of heavy reliance on C_4_ resources, probably millet (López‐Costas & Müldner, 2016). Millet (C_4_ plant) has been directly consumed by humans or, like *spartina sp*., used as fodder in the region of Catalonia since the 2nd millennium BC (Alonso Martínez, 2000; López‐Costas & Müldner, 2016; Tafuri et al., 2009). The fact that this is the only outlier could suggest that this male individual came from an area where C_4_ plants were more widely consumed. Alternatively, this individual may have been local and simply had a diet more reliant in this type of resources. It may be possible in the future to investigate the migration of this individual by measuring other isotopic systems such as strontium or oxygen in dental enamel.

### 4.2 An isotope signature for DISH?

Most of the bioarchaeological studies that are focused on the prevalence of DISH note the relationship between DISH and a high protein, high trophic level diet, potentially associated with high status individuals or monastic communities (Giuffra et al., 2010; Jankauskas, 2003; Rogers & Waldron, 2001; Verlaan et al., 2007; Waldron, 1985). Therefore, it is notable that, besides the work published here, only Müldner and Richards (2007a), Spencer (2008) and Quintelier et al. (2014) have investigated the relationship between DISH and diet using carbon and nitrogen stable isotope analysis. Müldner and Richards (2007a) reported on the *δ* ^13^C and *δ* ^15^N of four male individuals with DISH from Fishergate house. Their results show that, while individuals with DISH plotted with most of the males (*n* = 115), all individuals with DISH plotted above the population mean. The authors interpreted this as to suggest that their isotope values suggested a high‐protein diet with a significant inclusion of marine resources (Müldner & Richards, 2007a).

Spencer (2008) analyzed eight monastic and non‐monastic communities (total sample size = 46; DISH = 23, non‐DISH = 23; male = 40, female = 6). In this analysis, the individuals with DISH had statistically higher *δ* ^15^N compared to the individuals without DISH (13.4‰ and 12.7‰, respectively), but the difference from the mean *δ* ^13^C was not statistically significant. However, this difference only exists when the entire population or the monastic population is considered but does not hold true when only the male subsample is considered. No significant difference between sexes was found in the *δ* ^15^N in either the DISH or the non‐DISH subsamples (Spencer, 2008). Spencer (2008) noted that despite the statistically significant difference in *δ* ^15^N between individuals with and without DISH, the *δ* ^15^N values for individuals with DISH were not consistently higher compared to those obtained for individuals without DISH. This is because some individuals with DISH showed lower *δ* ^15^N values compared to individuals without DISH. Still, the author stressed that the trend was for the individuals with DISH to show higher *δ* ^15^N than individuals without DISH. Spencer (2008) concluded that dietary differences between these two groups did, in fact, exist and possibly consisted in the individuals with DISH following a diet with a higher intake of animal protein and of foods of a higher trophic level possibly of terrestrial origin (e.g., omnivore protein, freshwater fish, or a mix of freshwater and marine resources) as the *δ* ^13^C still suggested a terrestrial diet.

Finally, Quintelier et al. (2014) reported on the study of the relationship between DISH and diet based on the remains from the Carmelite friary in Aalst, Belgium. Their results showed that while males with DISH had slightly higher *δ* ^13^C and *δ* ^15^N compared to the individuals without DISH, these differences were not statistically significant. They argued that these results were possibly linked to the small sample size (15 males and 14 females without DISH, and 10 males with DISH). Quintelier and colleagues analyzed collagen extracted from ribs and from the bony lesions themselves to test whether pathological lesions showed isotopic ratios that were independent of diet. As no differences were found between the two types of samples, the authors suggested that DISH was not a pathological condition that influenced the bone *δ* ^13^C and *δ* ^15^N values (Quintelier et al., 2014). Furthermore, these data show that the diet must have been isotopically similar both before and during the period of formation of the pathological bone.

While the difference in the prevalence of DISH between the samples from Baldock and Santa Caterina is not significant, the former had a higher percentage of sample affected by DISH. It is, however, Santa Caterina that shows higher *δ* ^13^C and *δ* ^15^N. These results, therefore, suggest that higher *δ* ^15^N need not to be related to an increased predisposition to develop DISH, as these *δ* ^15^N values could be related to an increased intake of freshwater resources that would not lead to the metabolic imbalances associated to obesity and, in turn, DISH.

This is the first research that has considered the progressive nature of DISH when investigating its relationship with diet. DISH is a slow progressing disease which takes at least a decade of development to affect the four vertebrae usually required for its diagnosis (Mader, 2008; Yaniv et al., 2014). With such a lengthy development process, if DISH were caused by “rich diet,” it is expected that this would have been reflected in higher *δ* ^13^C and *δ* ^15^N. However, there is no discernible pattern of higher *δ* ^13^C and *δ* ^15^N in individuals with more advanced disease, in fact, the individuals from both archaeological sites with Stage 1 of DISH development show higher *δ* ^15^N and similar *δ* ^13^C than the individual in stage 2, and the *δ* ^15^N of the individuals from Baldock with Stage 4 is marginally higher than those with Stage 1 and 2. Therefore, these results suggest that there is no correlation between severity of DISH and *δ* ^13^C and *δ* ^15^N.

Considering previous studies and the results obtained in this project, it is clear that there is no evidence for an isotopically different diet between individuals with and without DISH. Furthermore, as Spencer (2008: 254) argued, even when there might be a significant difference in *δ* ^15^N, it is possible that this difference is not related to the dietary pattern but that it reflects physiological processes; as variation in *δ* ^15^N has also been found to be related to growth, illness and physiological stress (Beaumont et al., 2015; D'Ortenzio et al., 2015; Katzenberg & Lovell, 1999; Waters‐Rist & Katzenberg, 2010). Assuming that there is a relationship between DISH and social status, it is also possible that the dietary differences were not reflected on the isotope signature because the types of food consumed by the different social groups were isotopically similar. In fact, wealth may have been reflected in the types of meat consumed and the ability to afford more diverse and exclusive foods (e.g., goat, pig and/or marine fish depending on the time period and the region) that could have little impact on the overall dietary *δ* ^13^C and *δ* ^15^N (e.g., garum, wild game) (Fàbrega, 2016; Mays, 1997; Müldner & Richards, 2005; Müldner & Richards, 2007a, 2007b; Redfern et al., 2010; Thibaut i Comalada, 2006, pp. 38, 59). It should be considered that the rich diet linked to the development of DISH has usually been translated to mean high‐meat consumption; however, some marine species as well as dairy products are also protein‐rich and are known to have been consumed throughout time. And finally, it should be borne in mind that the development of DISH is most likely associated to a group of predisposing factors, such as obesity and metabolic imbalances, which may or may not include diet.

Stable isotope analysis data from the Romano‐British site of Baldock and the Roman Catalan site of Santa Caterina suggest that these populations followed a slightly different diet once the faunal data is considered. The individuals from Baldock mainly consumed terrestrial resources and the individuals from Santa Caterina consumed a combination of terrestrial and fish resources. In addition, the animals and one human from Santa Caterina consumed C_4_ plants. With regards to the investigation of dietary characteristics in individuals with and without DISH, it was hypothesized that if individuals with DISH followed a richer diet (i.e., higher consumption of meat and fatty fish), they would also show higher *δ* ^13^C and *δ* ^15^N. One of the limitations of this study is the small sample size of individuals; nonetheless, the *δ* ^13^C and *δ* ^15^N obtained from the individuals with DISH are very similar to those obtained from the individuals without DISH. In addition, the elevated *δ* ^15^N seen in the Santa Caterina population did not result in a higher prevalence of DISH or early DISH lesions. It is nevertheless worth noting that the development of DISH is, most probably, multifactorial and that individual characteristics might have influenced the development of the condition. The data presented in this paper shows that while DISH may have been influenced by individual's dietary habits, this cannot be investigated using isotope analysis. As this research has focused only on urban settlements, future research should focus on investigating DISH as a multifactorial disease and therefore considering age, sex, social status and access to resources, and ways of life (i.e., monastic and lay, rural and urban).

## CONFLICT OF INTEREST

The authors declare no potential conflict of interest.

## AUTHOR CONTRIBUTIONS

**Laura Castells Navarro:** Conceptualization (lead); formal analysis (lead); investigation (lead); methodology (lead); writing – review and editing (lead). **Jo Buckberry:** Conceptualization (equal); methodology (supporting); supervision (lead); visualization (supporting); writing – review and editing (equal). **Julia Beaumont:** Conceptualization (supporting); methodology (supporting); supervision (supporting); visualization (supporting); writing – review and editing (equal).

## Supporting information


**Table S1**: Isotope data from Romano‐British sites in England.
**Table S2**: Isotope data from Roman sites in the Mediterranean area.

## Data Availability

The data that supports the findings of this study are available in the tables and supplementary material of this article.
