# Supplementary material for: An isotope signature for diffuse idiopathic skeletal hyperostosis?
Source: Am J Biol Anthropol. 2022 Mar 3;178(2):312–27. doi: 10.1002/ajpa.24497 (PMC9313887; doi:10.1002/ajpa.24497)
Supplement: Supplementary file 1 — Table S1: Isotope data from Romano‐British sites in England. Table S2: Isotope data from Roman sites in the Mediterranean area. [file AJPA-178-312-s001.docx]

**Supplementary Table 1: Isotope data from Romano-British sites in England**

| **Reference** | **Location** | **Period** | **Rural/urban** | **Sample size** | **δ^13^C** | **δ^15^N** | **Comment** |
| --- | --- | --- | --- | --- | --- | --- | --- |
| Redfern et al., 2010 | Dorchester | RB | Mean | 32 | -19.4±0.59 | 9.4±0.92 |  |
|  | Alington Avenue G1 | RB | Rural | 6 | -19 | 10.8 |  |
|  | Alington Avenue G2 | RB | Rural | 6 | -19.5 | 9 |  |
|  | Maiden Castle Road | RB | Rural | 5 | -19.8 | 9 |  |
|  | Albert Road | RB | Urban | 5 | -19.6 | 10 |  |
|  | Old Vicarage | RB | Urban | 6 | -19.2 | 9.8 |  |
| Fuller et al., 2006 | Queensford Farm | RB |  | 16 | -19.5±0.4 | 10.6±0.5 | Males |
|  |  | RB |  | 16 | -19.7±0.3 | 9.9±0.9 | Females |
| Müldner and Richards 2007 | Trentholme Drive, York | RB | Urban | 42 | -19.4±0.6 | 11.1±0.9 |  |
|  | Blossom Street, York | Late RB | Urban | 17 | -19.4±0.6 | 11.3±0.5 |  |
| Chenery et al., 2011 | Catterick | RB | Urban | 48 | -20.1±0.5 | 10.8±0.7 |  |
| Lightfoot et al., 2009 | Yarnton, Oxfordshire | RB | Rural | 5 | -19.6±0.3 | 11±2 |  |
| Richards et al., 1998 | Poundbury Camp | LIA-ERB |  | 13 | -19.9 | 8.5 |  |
|  |  | Late RB |  | 10 | -18.2 | 10.1 | Wealthy |
|  |  | Late RB |  | 21 | -19.5 | 9.3 |  |
|  |  | Post-RB |  | 1 | -19.9 | 7.3 |  |
| Cheung et al., 2012 | Gloucester | RB | urban | 32 | -19.8 | 10.5 |  |
|  | Cotswold Community | RB | Rural | 24 | -20.5 | 9.8 |  |
|  | Horcott Quarry | RB | Rural | 22 | -20.1 | 9.3 |  |

**Supplementary Table 2: Isotope data from Roman sites in the Mediterranean area**

| **Reference** | **Location** | **Period** | **Rural/urban** | **Sample size** | **δ^13^C** | **δ^15^N** | **Comments** |
| --- | --- | --- | --- | --- | --- | --- | --- |
| Rissech et al., 2016 | Barcelona, Spain | Roman (1st-4th AD) | Urban | 24 | -18.8±0.4 | 11.0±0.4 | Males |
|  |  |  |  | 11 | -19.0±0.2 | 11.1±0.5 | Females |
|  |  |  |  | 11 | -18.9±0.3 | 11.1±0.4 | Average |
| Lópex-Costas & Müldner 2016 | A Lanzada, Galicia, Spain | Roman | Rural | 43 | -16.7±1.0 | 12.1±0.9 |  |
|  |  | Post Roman | Rural | 15 | -14.3±0.7 | 12.8±0.5 |  |
| Fuller et al., 2010 | Ibiza | 4th-6th centuries | Urban | 60 | -19.0±0.4 | 11.1±1.1 |  |
| Garcia et al., 2004 | Mallorca | Late Roman 600AD | Urban | 30 | -18.7 | 10.6 |  |
| Lightfoot et al., 2012 | Zadar-Reljie (Croacia) | Roman | Urban | 51 | -18.9±0.4 | 10.0±0.7 |  |
|  | Vis-Bandirica (Croacia) | Roman (island) |  | 14 | -18.9±0.3 | 9.7±0.7 |  |
|  | Podvršje (Croacia) | Late Roman | Rural | 11 | -18.5±0.3 | 10.9±0.9 |  |
|  | Croacia | - | - | 76 | -18.8±0.4 | 10.1±0.8 |  |
| Keenleyside et al., 2009 | Leptiminus - Tunisia | Roman | Urban | 16 | -17.7±0.7 | 13.0±1.3 | Males |
|  |  |  |  | 21 | -17.8±0.5 | 13.0±1.5 | Females |
|  |  |  |  | 99 | -17.7±0.6 | 13.4±1.8 | Average |
| Craig et al., 2009 | Velia - Italy | Roman | Urban | 100 | -19.5±0.2 | 8.2±0.7 | Group I |
|  |  |  |  | 17 | -19.3±0.3 | 11.2±1.3 | Group II |
| Prowse et al., 2004, 2005 | Isola Sacra - Italy | Roman | Urban | 48 | -18.7±0.3 | 11.0±1.1 | Males |
|  |  |  |  | 32 | -18.8±0.3 | 10.6±1.0 | Females |
|  |  | Roman Imperial | Urban | 105 | -18.8±0.3 | 10.8±1.2 | Average |
| Fuller et al., 2012 | Sagalassos - Turkey | Classical-Hellenistic (400-200 BC) | Urban | 4 | -19.4±0.4 | 9.7±0.7 |  |
|  |  | Late Imperial (300-450 AD) | Urban | 3 | -19.2±0.2 | 10.1±0.7 |  |
